# PhenPath: a tool for characterizing biological functions underlying different phenotypes

**DOI:** 10.1186/s12864-019-5868-x

**Published:** 2019-07-16

**Authors:** Giulia Babbi, Pier Luigi Martelli, Rita Casadio

**Affiliations:** 1 University of Bologna, FABIT, Via San Donato 15, 40126 Bologna, Italy; 2 Department of BIGEA, University of Bologna, Piazza di Porta S. Donato, 1, 40126 Bologna, Italy; 3 Interdepartmental Center “Luigi Galvani” for integrated studies of Bioinformatics, Biophysics and Biocomplexity, University of Bologna, CIG, Via G. Petroni 26, 40126 Bologna, Italy; 4 CNR, Institute of Biomembrane and Bioenergetics (IBIOM), Via Giovanni Amendola 165/A, 70126 Bari, Italy

**Keywords:** Phenotype, Diseases, Molecular pathway, Biological process, Enrichment

## Abstract

**Background:**

Many diseases are associated with complex patterns of symptoms and phenotypic manifestations. Parsimonious explanations aim at reconciling the multiplicity of phenotypic traits with the perturbation of one or few biological functions. For this, it is necessary to characterize human phenotypes at the molecular and functional levels, by exploiting gene annotations and known relations among genes, diseases and phenotypes. This characterization makes it possible to implement tools for retrieving functions shared among phenotypes, co-occurring in the same patient and facilitating the formulation of hypotheses about the molecular causes of the disease.

**Results:**

We introduce PhenPath, a new resource consisting of two parts: PhenPathDB and PhenPathTOOL.

The former is a database collecting the human genes associated with the phenotypes described in Human Phenotype Ontology (HPO) and OMIM Clinical Synopses. Phenotypes are then associated with biological functions and pathways by means of NET-GE, a network-based method for functional enrichment of sets of genes. The present version considers only phenotypes related to diseases. PhenPathDB collects information for 18 OMIM Clinical synopses and 7137 HPO phenotypes, related to 4292 diseases and 3446 genes. Enrichment of Gene Ontology annotations endows some 87.7, 86.9 and 73.6% of HPO phenotypes with Biological Process, Molecular Function and Cellular Component terms, respectively. Furthermore, 58.8 and 77.8% of HPO phenotypes are also enriched for KEGG and Reactome pathways, respectively. Based on PhenPathDB, PhenPathTOOL analyzes user-defined sets of phenotypes retrieving diseases, genes and functional terms which they share. This information can provide clues for interpreting the co-occurrence of phenotypes in a patient.

**Conclusions:**

The resource allows finding molecular features useful to investigate diseases characterized by multiple phenotypes, and by this, it can help researchers and physicians in identifying molecular mechanisms and biological functions underlying the concomitant manifestation of phenotypes. The resource is freely available at http://phenpath.biocomp.unibo.it.

**Electronic supplementary material:**

The online version of this article (10.1186/s12864-019-5868-x) contains supplementary material, which is available to authorized users.

## Background

Co-occurrence of different phenotypes often associated with symptom complexes hampers the understanding of the molecular mechanisms which characterize diseases and their insurgence [1]. Furthermore, the analysis of epidemiological data reveals that different phenotypes associated with specific diseases frequently co-occur in the same individuals during their lifespan [2, 3]. In both situations, highlighting functional molecular mechanisms underlying disease insurgence and progression offers a way to understand possible associations between phenotypes and diseases. In the context of personalized medicine, this approach can be in principle adopted to analyze phenotypes that are peculiar of every single patient. The challenge is to reconcile the ensemble of phenotypes with a small number of possibly altered biological functions. Along this line, Brodie et al. (2014), [4], reported a large-scale analysis of Genome Wide Studies (GWAS) results demonstrating that phenotypes can be significantly associated to specific pathways, where SNPs cluster, depending on the specific disease.

Several resources are presently available to exploit data for associating phenotypes to diseases. The Phenotype-Genotype Integrator (PheGenI) [5] merges data from genome-wide association study (GWAS) stored at the National Human Genome Research Institute (NHGRI) with several databases housed at the National Center for Biotechnology Information (NCBI), including Gene, dbGaP, OMIM, eQTL and dbSNP. This phenotype-oriented resource aims at facilitating prioritization of variants from GWAS studies, for generation of biological hypotheses and it is quite useful for a search based on chromosomal location, gene, SNP, or phenotype. Search results include annotated tables of SNPs, genes and association results, a dynamic genomic sequence viewer, and gene expression data.

For the molecular diagnosis of rare genetic diseases, the recently developed Phenopolis [6] is an open platform for harmonization and analysis of sequencing and phenotype data. The platform offers, for each phenotype, a prioritized list of genes, based on known association and gene enrichment analysis.

Other resources provide associations between diseases and phenotypes, including the Human Phenotype Ontology (HPO) [7] and the OMIM Clinical synopses [8]. Exploiting these associations, methods have been developed to cluster different diseases through shared phenotypes. In particular, the Phenotypic Disease Network [9] focuses on phenotypic links among co-occurring diseases to address the comorbidity problem. The Phenomizer tool [10], provided by the Human Phenotype Ontology consortium, analyzes lists of phenotypes/symptoms with the aim of assisting the clinical workflow and providing diagnoses.

While many resources focus on the relationship among phenotypes, diseases and genes, little is known about the relevance of molecular functions and functional processes underlying the occurrence of phenotypes.

The goal of our research is to supplement disease-phenotype associations with information at the molecular level. To this aim, here we describe a resource (PhenPath) able to retrieve diseases, genes and functional annotations associated with a given set of phenotypes.

Our resource builds on supplementing known disease-phenotype links with the molecular information on the association between genes and diseases. This last knowledge is stored in different databases, including Humsavar [11], ClinVar [12] and OMIM [8], previously integrated by DisGeNet [13] and by eDGAR [14], which exploits also functional annotations.

Phenotype-disease and disease-gene relationships can be represented with a graph and, after collapsing the disease layer, direct associations between genes and phenotypes emerge. Furthermore, efficient enrichment procedures help in associating groups of genes to specific biological processes and/or metabolic pathways, endowing the group with statistically validated functional annotations. Among other procedures, our NET-GE [15] exploits proximity relationships among genes as derived from gene-gene interaction networks [16], and here it is adopted to functionally annotate phenotype-related genes. Considering the relationship among diseases, genes and functions, and the association among diseases and phenotypes, PhenPath allows the association of phenotypes to biological processes and pathways, reconciling their manifestation with molecular events.

## Results

We implemented a new resource, PhenPath, to help researchers and physicians in studying complex diseases, characterized by one or multiple phenotypes.

PhenPath consists of two parts: a database collecting relationships among genes, diseases, phenotypes and biological functions (PhenPathDB), and a tool allowing to retrieve genes, diseases and biological functions shared by a group of phenotypes, provided by the user (PhenPathTOOL).

### PhenPathDB

PhenPathDB is generated considering the three main steps described in the following: i) a phenotype-disease association procedure; ii) a disease-gene association procedure; and iii) a phenotype functional annotation derived by collapsing the gene layer, after an enrichment procedure of the functional annotation of the different disease-associated genes. Functional annotations consider Gene Ontology [17] terms of the three main roots (Molecular Function, Biological Process and Cellular Component), KEGG [18] and Reactome [19] pathways.

#### Phenotype-disease association

PhenPathDB builds upon the known associations among phenotypes, diseases and genes*.* PhenPathDB includes information about the following phenotypic terms (Table 1): i) 18 phenotypic general categories from the OMIM Clinical Synopsis [8], which classifies 4165 OMIM diseases, grouped according to the affected human body districts; ii) 7173 phenotypic terms from HPO [7], annotating 4292 OMIM diseases (59% of the 12,111 phenotypic terms of HPO, which are disease-associated). HPO includes five main sub-ontologies (Phenotypic Abnormalities, Clinical Modifier, Clinical Course, Mode of Inheritance, and Frequency). Specific terms, called leaf terms, are 3837 and they annotate at the deepest level 4023 diseases. The most populated sub-ontology is Phenotypic Abnormalities, which includes 78% of the HPO disease-related phenotypes with 24 main categorizations referring to body districts and physiological functions. They expand into 5661 terms associated with 4273 diseases, of which 3802 are leaf terms annotating 3721 diseases (Table 1).

**Table 1:** Phenotypic terms included in PhenPathDB

Ontology	Phenotypic terms (#)	Diseases associated to Phenotypic terms (#)	Phenotypic leaf terms (#)	Diseases associated to Phenotypic leaf terms (#)
OMIM Clinical Synopsis^a^	18	4165	–	–
HPO	7173	4292	3837	4023
HPO sub-ontology Phenotypic Abnormalities^b^	5661	4273	3802	3721

^a^OMIM Clinical Synopsis is not organized in a graph and, as a consequence, it does not contain distinction among root, intermediate and leaf terms. ^b^HPO Phenotypic Abnormalities are the subset of HPO, organized according to body districts and physiological functions into 24 different main terms

**Fig. 1:**
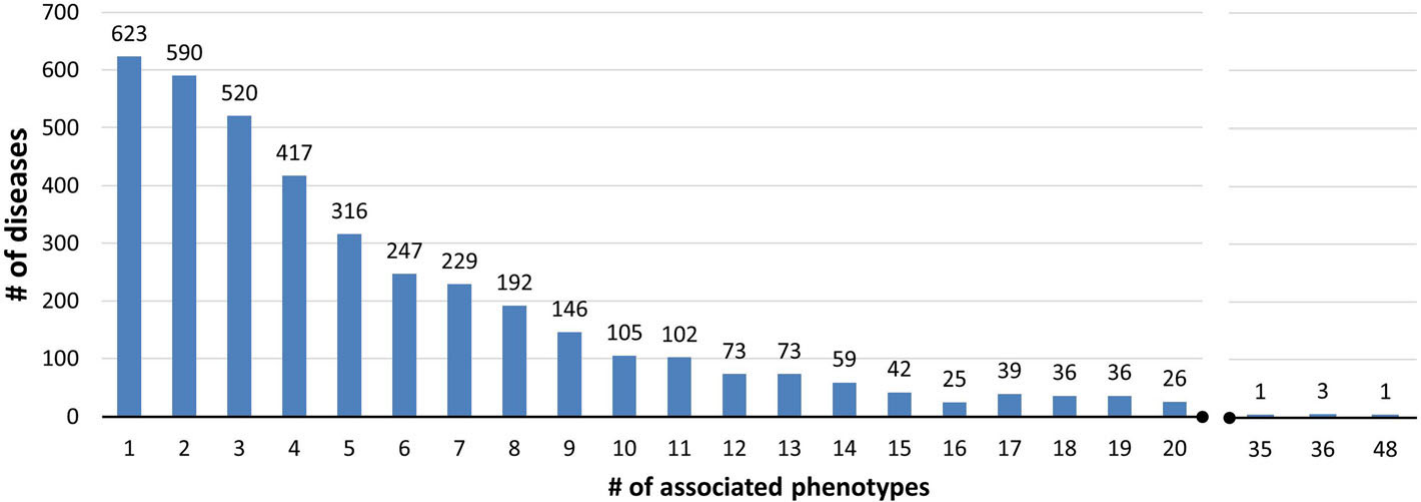
OMIM diseases as a function of associated HPO phenotypes. Data include 3837 HPO phenotypes (leaves of the HPO ontology) associated with 4023 OMIM diseases (Table 1, second row). Only 623 diseases (15%) are associated with a single phenotype, while about half of the diseases (47%) are associated with 5 or more phenotypes. Rubinstein-Taybi syndrome has the maximum number of associated HPO phenotypes (48, considering only leaves of the HPO graph)

Most of the OMIM diseases are associated with more than one HPO leaf term (Fig. 1). Only 15% of diseases are associated with one phenotype, and about half of the diseases are associated with 5 or more phenotypes. The extreme case is the Rubinstein-Taybi syndrome that is annotated with 48 HPO leaf terms.

#### Disease-gene association

**Fig. 2:**
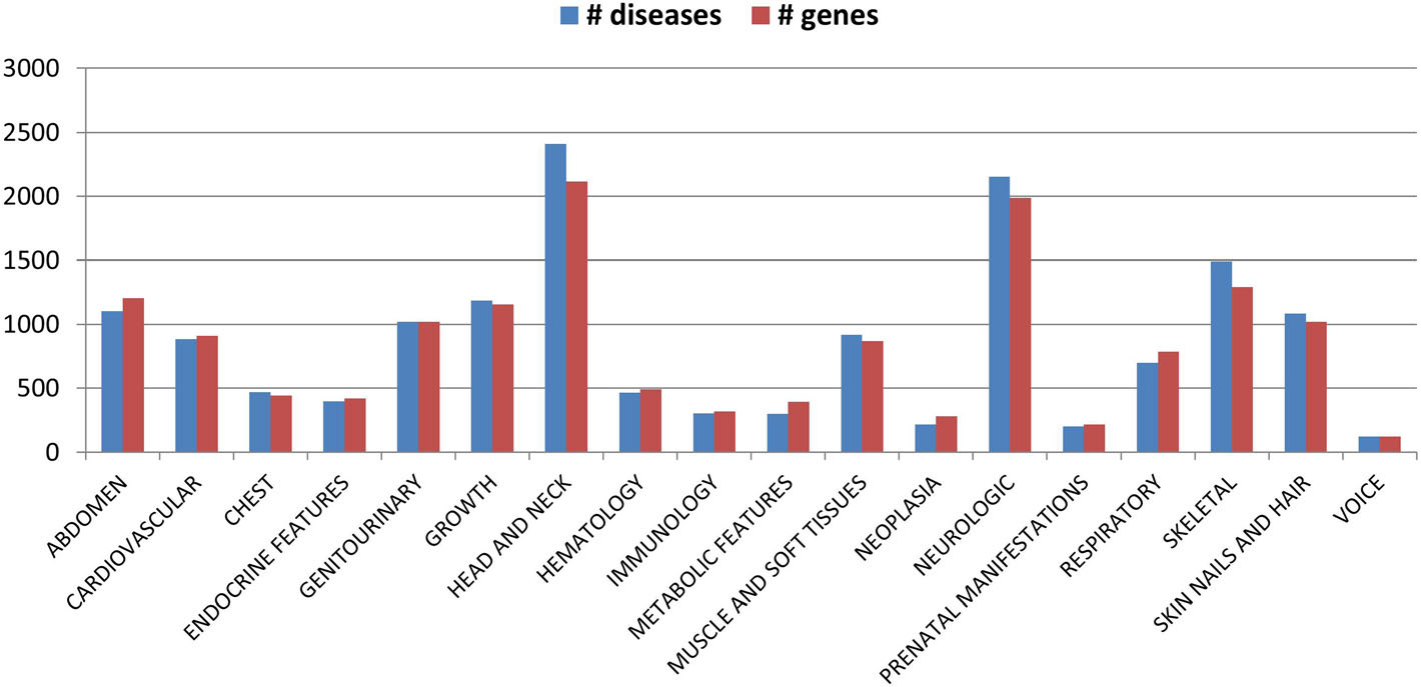
Number of diseases and genes associated with OMIM Clinical Synopsis terms. Blue bars (diseases); red bars (genes)

**Fig. 3:**
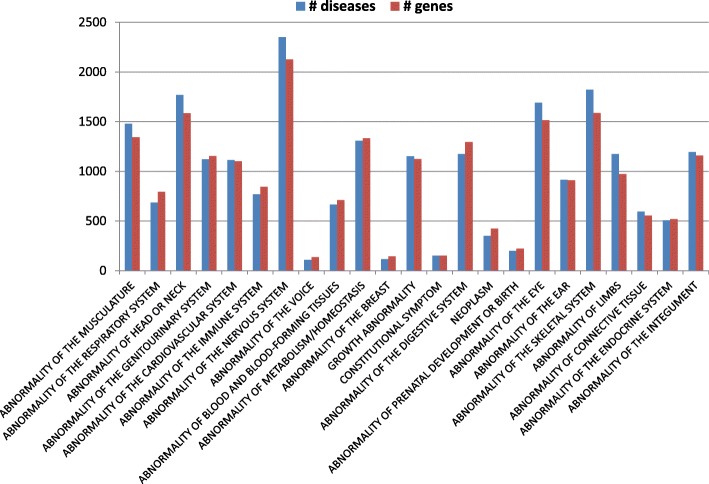
Number of diseases and genes associated with HPO Phenotypic Abnormalities sub-ontology. The 24 roots refer to anatomic districts and physiological functions. Blue bars (diseases); red bars (genes)

Each phenotype-disease link described in the previous section is supplemented with a set of genes, by exploiting the gene-disease relationships reported in eDGAR [14]. Figures 2 and 3 show the number of diseases (blue bars) and genes (red bars) associated to the 18 terms of the OMIM Clinical Synopsis and to the 24 main categories of the HPO Phenotypic Abnormalities sub-ontology, respectively. With eDGAR, Phenotypic OMIM Clinical Synopsis terms and HPO terms are associated with 3230 and 3446 genes, respectively.

#### Functional annotation of phenotypes

According to our procedure, any phenotype links one or more disease/s, which are associated with specific genes. Any set of genes can be functionally characterized by adopting an enrichment procedure. Here, we adopt NET-GE, a tool for the functional enrichment analysis of genes (two or more) [15]. NET-GE considers the relationships among annotated genes as described in the STRING interactome, from which it derives a function-specific gene module to be used as a basis for the overrepresentation analysis. This procedure takes into consideration Gene Ontology terms, KEGG and Reactome pathways.

Following enrichment, most phenotypes included in Table 1, are annotated with Gene Ontology (GO) terms, as shown in Table 2. In particular, 87.7% and 86.9% of HPO terms are enriched with GO terms of Biological Process (BP) and Molecular Function (MF), respectively.

**Table 2:** Functional annotation of HPO terms

Functional Annotation	Phenotypes (#)	HPO terms (%)	Non redundant functional terms (#)
with GO BP	6256	87.7%	6838 GO BP
with GO MF	6202	86.9%	2211 GO MF
with GO CC	5254	73.6%	946 GO CC
with KEGG	4198	58.8%	326 KEGG
with REACTOME	5550	77.8%	1369 REACTOME

Statistics refer to 7137 HPO terms comprised in PhenPath and associated with 4292 diseases and 3446 genes. Terms included in PhenPath comprise 59% of the 12,111 terms listed in HPO. *BP* Biological Process, *MF* Molecular Function, *CC* Cellular Component, *#* number of

### PhenPathDB interface

PhenPathDB organizes associations among phenotypes, diseases, genes and functional annotations in two major entering tables: OMIM Clinical Synopsis and HPO Phenotypic Abnormality (http://phenpath.biocomp.unibo.it). Each Table contains links to our results grouped into:

i). *general analysis*, which, for each phenotype*,* lists diseases, associated genes and the functional characterization derived from the enrichment procedure;
ii). *intersection analysis*, which allows to derive features shared between two phenotypes, highlighting the common diseases, genes and functional annotations.

More specifically, *general analysis* reports diseases and genes associated with the phenotype, the annotation obtained with NET-GE, along with the Bonferroni-corrected *p*-value of the enrichment procedure, and the Information Content (IC) evaluating the specificity of the term (see Methods section for further details). The page lists also the genes accounting for the enrichment of each functional term and the associated diseases, to describe the association of specific functional terms with the phenotype under consideration. Diseases and genes are linked to the corresponding OMIM and Human Gene Nomenclature Committee (HGNC) [20] entries.

The *intersection analysis* is based on the pre-computed shared features of pairs of phenotypes out of the same ontology (18 categories of OMIM Clinical Synopsis or 24 main categories of HPO Phenotypic Abnormalities sub-ontology). Furthermore, shared GO terms, KEGG and Reactome pathways, enriched for both groups of associated genes, are listed. For each functional term, the IC value is reported as well as the Bonferroni-corrected *p*-values of the two enrichment procedures. The phenotype page provides also the list of genes associated with a particular functional term.

It is possible to access the database either by browsing the PhenPathDB page or by searching for specific phenotypes in the Search page. For HPO, the 24 main categories of the Phenotypic Abnormalities are present in the browsing page, and all terms can be retrieved with a search.

### PhenPathTOOL

PhenPathTOOL is a web application that, given a set of phenotypes, retrieves the shared diseases, genes and functional terms. PhenPathTOOL is user-friendly, accepting as input HPO IDs as well as names of phenotypes. The intersection is computed in real-time. PhenPathTOOL allows investigating the relationship among groups of phenotypes at different levels. Firstly, it retrieves whether there is an intersection among the lists of diseases associated with the input phenotypes. In this way, it highlights when the phenotype co-occurrence is already known and points towards specific diseases. Occasionally, when input phenotypes do not share common diseases, PhenPathTOOL can retrieve shared genes, possibly related to their concomitant manifestation*.* Furthermore, even when phenotypes do not share genes, they may share the enriched biological functions (GO terms, KEGG and Reactome pathways), accounting for a common mechanism. The interface reports in different tables the lists of shared GO terms, KEGG and REACTOME pathways, obtained as described above. Each table lists the IC of the term, as well as the Bonferroni-corrected *p*-value for each association (see Methods for further details).

## Discussion

### Case Study: Tourette syndrome

The first example describes the use of PhenPathTOOL for retrieving a characterized disease starting from a list of phenotypes. It also illustrate the possibility to enrich the annotation of involved biological functions. Tourette syndrome is a neurobehavioral disorder that causes motor and vocal tics associated with behavioral abnormalities, like attention-deficit–hyperactivity disorder and obsessive-compulsive disorder [21]. Possible symptoms include involuntary or semi-voluntary movements or sounds, repetitive movements, blinking, nose twitching, throat clearing to echolalia or coprolalia.

**Fig. 4:**
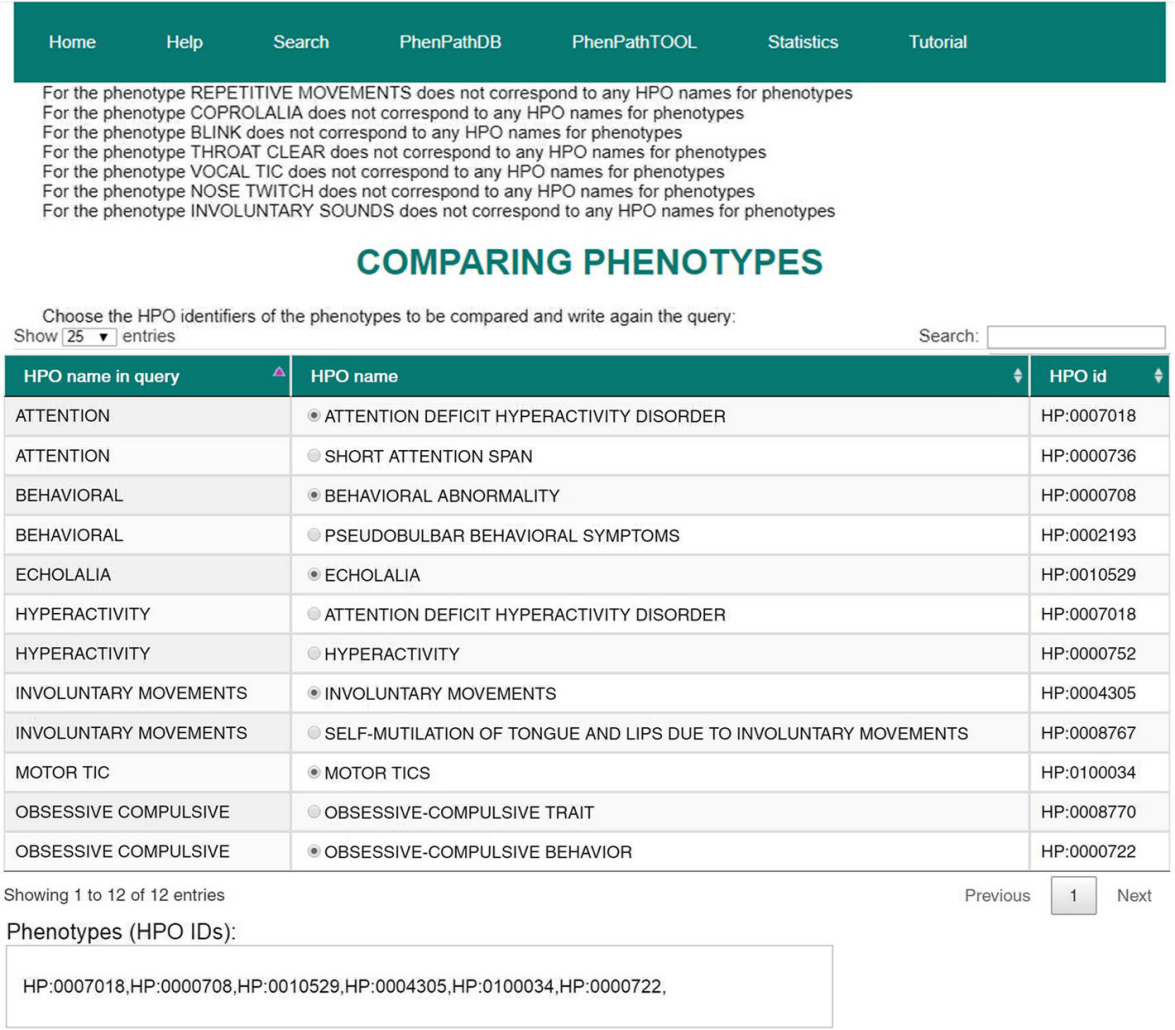
Selection of phenotypes in PhenPathTOOL. After searching with a list of different names, the web interface shows all the names that do not correspond to any HPO identifier and then a table with all the HPO terms matching the input. The user may then select the most appropriate phenotypes to be analyzed

We searched with PhenPathTOOL the typical phenotypic traits of the Tourette syndrome, using a plain list of phenotype names (“motor tic, vocal tic, behavioral, attention, hyperactivity, obsessive-compulsive, involuntary movements, involuntary sounds, repetitive movements, blink, nose twitch, throat clear, echolalia, coprolalia”). The interface presents a selectable list of HPO terms whose names contain the input terms (Fig. 4).

**Fig. 5:**
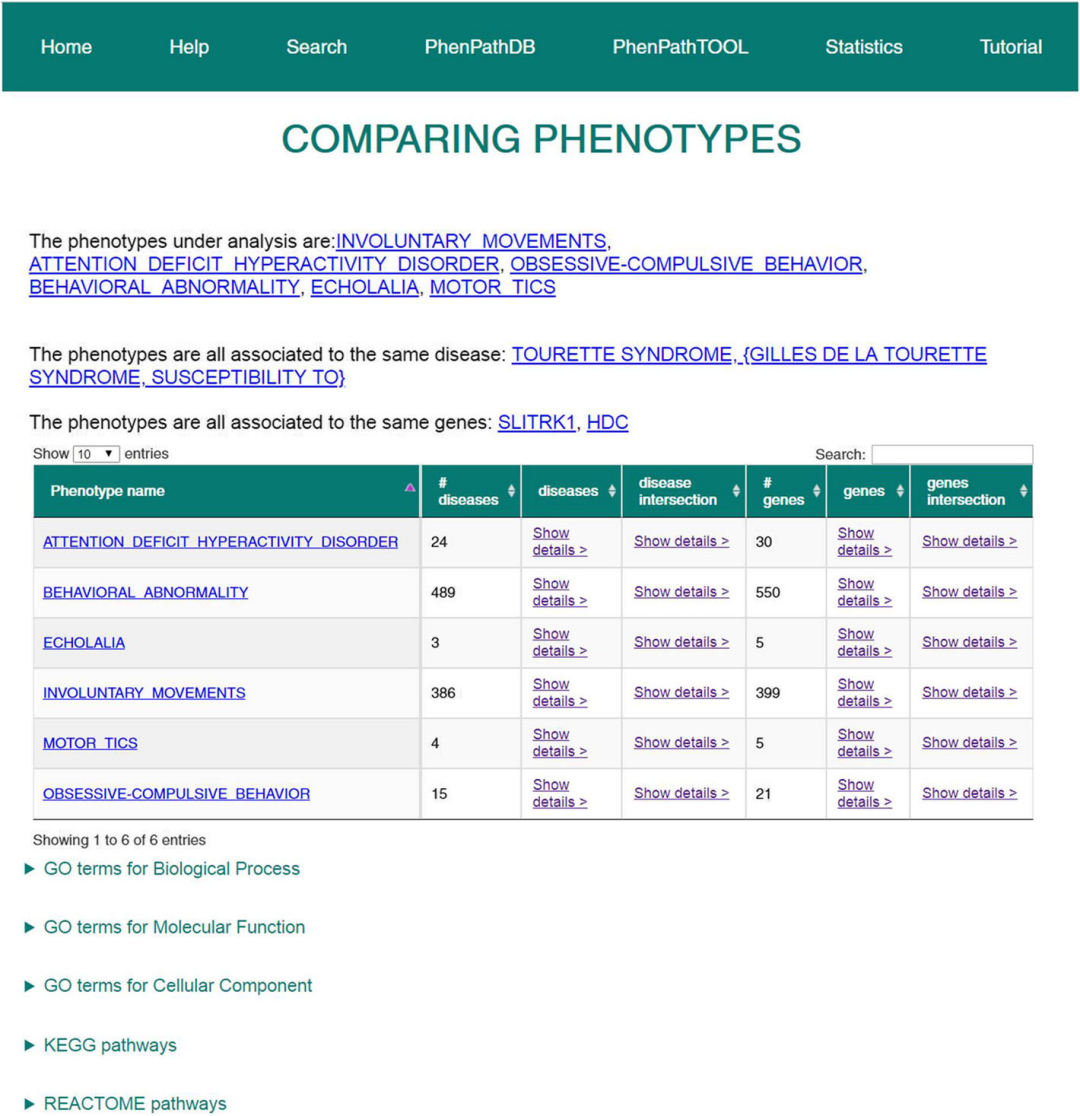
PhenPathTOOL results. The figure shows the webpage of PhenPathTOOL after the analysis of 6 different HPO phenotypes. First, a list of the shared diseases and genes is reported. Then, a general table collects data on diseases and genes associated with each phenotype, allowing direct intersection. The last section reports the links to the analysis of GO terms, KEGG and Reactome pathways

As shown in Fig. 5, PhenPathTOOL correctly recognizes that the concomitance of phenotypes points to the Tourette syndrome, and to two genes (SLITRK1, HDC) that are associated with the disease [21]. Interestingly enough, the intersection of functional terms shared by different phenotypes is able to retrieve relevant shared annotations. 30 terms are shared by at least 4 phenotypes. Among them, besides the general annotations like *behavior*, *cognition* or *learning or memory*, there are interesting clues on more specific pathways such as *catecholamine metabolic process*. Interestingly, symptomatic therapies for the Tourette syndrome involve the control of neurotransmission from dopamine and adrenaline, which are members of the catecholamine family [22]. Although the pathogenesis of the disorder remains obscure, the catecholamine metabolic process pathway has already been studied in relation to the Tourette syndrome [23].

### Case study: obesity, diabetes and ovarian cyst

Here PhenPathTOOL compares three phenotypes that, although not being related to a common disease, are often co-occurring: obesity, diabetes and ovarian cysts. Epidemiological studies report that women affected by polycystic ovarian syndrome, for which ovarian cysts is the main phenotypes, are often showing also obesity and diabetes phenotypes [24]. In particular, increasing evidence points to an increase of type 2 diabetes in women affected by polycystic ovarian syndrome [25].

We analyzed with PhenPathTOOLS the three co-occurring phenotypes: Obesity (HP:0001513), Diabetes Mellitus type II (HP:0005978) and Ovarian Cyst (HP:0000138). Routinely, the three terms refer to specific diseases: however, in HPO they indicate phenotypes associated to different disorders.

As expected, no disease is common to all the input phenotypes. Diabetes and obesity share 3 diseases: *Prader-Willi Syndrome*, *Morbid obesity and spermatogenic failure*, and *Microcephalic osteodysplastic primordial dwarfism, type II*. No disease links ovarian cysts to either obesity or diabetes.

The analysis at the gene level retrieves only one gene shared among the three phenotypes: PPARG, the Peroxisome proliferator-activated receptor gamma, a nuclear receptor involved in lipid uptake and adipogenesis. More genes are shared between pairs of phenotypes: NPP1, AKT2 between diabetes and obesity, HNF1A, INSR and PPP1R3A between ovarian cysts and diabetes, and PTEN between ovarian cysts and obesity.

A better characterization of the common ground of the three phenotypes comes from the analysis of shared functional annotations. 7 GO terms for molecular function are shared, being *hormone receptor binding* (GO:0051427) the most specific one (IC = 6.84). Moreover, 58 GO terms for biological process are shared, 16 of which with IC values greater than 5. These include *generation of precursor metabolites and energy* (GO:0006091), *energy derivation by oxidation of organic compounds* (GO:0015980), *cellular response to peptide hormone stimulus* (GO:0071375), *developmental process involved in reproduction* (GO:0003006), *response to peptide hormone* (GO:0043434), *cellular response to hormone stimulus* (GO:0032870), *response to hormone* (GO:0009725), *response to insulin* (GO:0032868), *regulation of growth* (GO:0040008). Each term is associated with the three phenotypes by means of many genes, including PPARG. On the overall, the annotation points towards phenomena associated with the response to hormones, in particular insulin. Specifically, the response to insulin is associated with each phenotype with corrected *p*-values of 1E-9, 0.04 and 0.005, respectively for Diabetes, Obesity and Ovarian Cyst.

The novelty with PhenPathTOOL is that the co-occurrence of the three phenotypes is ascribed to defects of the response to insulin. Interestingly, recent literature confirms that insulin resistance is a common background for both obesity and diabetes mellitus type 2 [26] and that insulin is a key factor also in the uptake of glucose by ovarian tissues during the menstrual cycle of some rodent, primate and ruminant species [27]. In particular, the link between metabolic disorders and cystic ovarian disease has been studied in animal models [28], specifically for the insulin resistance as a pathogenic factor. Our analysis is also supported by the finding that the activity of PPARG, the only gene shared among the three phenotypes under investigation, is sufficient for whole-body insulin sensitization [29].

### Case study: Rett syndrome

PhenPathTOOL can be adopted to endow a disease (described with a set of phenotypes) with novel links to genes and functional terms, retrieved by intersecting the sets of genes and functional terms associated with the single phenotypes in PhenPathDB. As a case study, we here apply PhenPathTOOL to the detection of new associations between genes and Rett syndrome (RTT). RTT is a neurodevelopmental disorder corresponding to two OMIM entries (#312750 and #613454) linked to genes MECP2 (encoding methyl CpG binding protein 2) and FOXG1 (encoding the forkhead box protein G1), respectively [30, 31]. RTT primarily affects females and it is characterized by loss of language and communication skills, microcephaly, learning impairment, coordination, and other brain functions. Affected girls may lose the use of their hands and begin making repeated hand-wringing, washing, or clapping motions. Atypical forms of RTT, not reported in OMIM, have been described in patients not carrying mutations on FOXG1 nor MECP2 and manifesting additional phenotypes such as breathing abnormalities, spitting or drooling, unusual eye movements, cold hands and feet, irritability, sleep disturbances, seizures and scoliosis [32, 33]. Recently, literature reported new genes associated with RTT, including cyclin-dependent kinase-like 5 (CDKL5), myocyte-specific enhancer factor 2C (MEF2C), and transcription factor 4 (TCF4) [34–36]. These associations are not yet reported in major databases and, consequently, they are not included in PhenPathDB. We tested the ability of PhenPathTOOL to recover these associations starting from the phenotype description. We entered 9 HPO terms, characterizing the classical and atypical RTT, namely *breathing dysregulation* (HP:0005957), *abnormality of coordination* (HP:0011443), *drooling* (HP:0002307), *irritability* (HP:0000737), *severe expressive language delay* (HP:0006863), *specific learning disability* (HP:0001328), *microcephaly* (HP:0000252), *scoliosis* (HP:0002650), and *sleep disturbance* (HP:0002360).

As a first step, PhenPathTOOL intersects the gene sets associated with the phenotypes. Although no gene is common to the nine phenotypes, 5 genes (MECP2, CDKL5, UBE3A, SLC2A1, SLC16A2) are shared among 5 phenotypes. MECP2 and CDKL5 have been previously reported [32, 36]. Interestingly, our analysis highlights the association with CDKL5, which is not present in PhenPathDB.

PhentPathTOOL then retrieves the intersection of GO terms, KEGG and Reactome pathways enriched for the different phenotypes. Focusing on GO BP, 440 terms are shared among two or more phenotypes. In particular, when restricting to terms with medium/high specificity (IC > 4.5), 12 enriched terms are common to 5 or more phenotypes. Among them, the seven terms listed in Table 4 describe biological processes that involve the two genes known to be related with RTT (MECP2 and FOXG1), as well as TCF4, that has been only recently associated with RTT (Table 3).

**Table 3:** A selection of GO BP terms shared by the phenotypes in input after enrichment procedure

GO BP term	IC value	# of associated phenotypes	Associated phenotypes	Related genes associated with RTT
*cellular component morphogenesis*	5.4	6	*microcephaly, sleep disturbance, scoliosis, breathing dysregulation, abnormality of coordination, specific learning disability*	TCF4
*Behavior*	5.23	6	*microcephaly, specific learning disability, sleep disturbance, scoliosis, abnormality of coordination, drooling*	MECP2
*cell projection organization*	4.95	6	*microcephaly, specific learning disability, scoliosis, breathing dysregulation, abnormality of coordination, sleep disturbance*	MECP2
*neurological system process*	4.65	6	*microcephaly, specific learning disability, scoliosis, abnormality of coordination, sleep disturbance, drooling*	FOXG1, MECP2
*system development*	4.75	5	*microcephaly, sleep disturbance, scoliosis, abnormality of coordination, severe expressive language delay*	TCF4, FOXG1, MECP2
*anatomical structure formation involved in morphogenesis*	4.69	5	*microcephaly, specific learning disability, scoliosis, breathing dysregulation, abnormality of coordination*	TCF4, FOXG1
*single-organism behavior*	5.67	5	*microcephaly, sleep disturbance, scoliosis, abnormality of coordination, drooling*	TCF4, MECP2

These findings illustrate the efficacy of PhenPathTOOL in linking a set of phenotypes to genes and functional annotations, which can be adopted for planning further experimental analysis.

The table reports some of the most interesting biological processes associated with the phenotypes given as input to PhenPathTOOL. For each term, the IC value is shown with the specific phenotype associations. Noticeably, TCF4 has been only recently associated with RTT [36].

### Study case: associating genes to uncharacterized diseases

We propose PhenPath as a resource for formulating hypotheses on the molecular mechanisms underlying the manifestation of concomitant phenotypes, in particular in case of non-well characterized diseases. Here we estimate the performance of PhenPathTOOL in retrieving relevant associations between groups of co-occurring phenotypes and possible causative genes, collecting from Orphanet [37] a blind set consisting of 87 diseases, not included in OMIM nor, consequently, used to build PhenPathDB. Orphanet associates these diseases with both HPO phenotypic terms and sets of possibly causative genes (see Methods section 5.3 for further details on the dataset).

We evaluate the efficiency of PhenPathTOOL in retrieving genes starting from the phenotypic characterization of diseases. For each disease in the blind set, we entered in PhenPathTOOL the Orphanet-associated HPO terms and we retrieved the corresponding lists of shared genes. We then compared the genes retrieved with PhenPathTOOL with the genes proposed by Orphanet.

For 61 diseases out of 87 (70%), PhenPathTOOL retrieves at least one of the genes associated by Orphanet. Overall, out of the 100 genes associated by Orphanet, 58 are recovered with PhenPathTOOL (58%). In particular for 2 diseases, *Pituitary stalk interruption syndrome* and *Hypothyroidism due to deficient transcription factors involved in pituitary development or function*, PhenPath retrieves 5 out of 7 and 5 out 5 Orphanet-associated genes, respectively.

A summary of all the results obtained for the external dataset is provided as supplementary material (see Additional file 1).

## Conclusions

PhenPath offers a new approach for investigating the molecular mechanisms leading to the correlated manifestation of different phenotypes. PhenPath may be used to explore the possible connections among different phenotypes co-occurring in a patient, offering new clues on the biological mechanisms that may explain its clinical conditions.

Four case studies show the potential use of PhenPath for retrieving diseases starting from a set of phenotypes, if existing, and/or for better characterize the functions and pathways possibly involved in the manifestation of different symptoms. We propose our resource for directing scientific efforts, helping the diagnosis and retrieving new possible associations among biological processes and diseases. We believe that biotechnologists, physicians and medical researchers may find PhenPath a useful resource of information, especially when studying complex and rare diseases.

## Methods

### Associations among phenotypes, diseases and genes

PhenPathDB stands on the merging of disease-phenotype and disease-gene relationships. In PhenPath, a phenotype is defined as an actual physical characteristic, and we follow the phenotype characterization provided by HPO and OMIM Clinical Synopsis. We define a disease as a medical condition associated with specific phenotypes and we classify diseases according to OMIM identifiers.

In detail, two lists of phenotype terms have been considered: the OMIM Clinical Synopsis (March 2017 release) and the HPO Phenotypic Abnormalities categories (May 2017 release). OMIM Clinical Synopsys groups OMIM diseases within 22 phenotypic categories, 18 referring to systems of the human body (e.g.: respiratory system, musculature, etc.) and 4 referring to further levels of characterization (inheritance, laboratory abnormalities, molecular basis, and miscellaneous). In PhenPath, we retained the former and discharged the others, ending up with the phenotypic characterization for 3230 diseases.

The HPO consists of 12,111 different phenotypes organized into a direct acyclic graph (DAG) including 3837 leaf phenotypes. A leaf in a graph is a node without sub-nodes (children), and, by consequence, a leaf phenotypic term provides the most detailed level of annotation. When a phenotype is associated with a disease by HPO, the annotation is extended to all the parent phenotypes in the HPO DAG. On the overall, 4292 OMIM diseases are associated with 7137 HPO phenotypes, which represent the 59% of all the HPO phenotypes. In particular, 4023 diseases are associated with 3837 leaf phenotypes. Of particular interest are the phenotypes originating from 24 main categories, referring to human body districts and physiological functions (musculature, respiratory system, head or neck, genitourinary system, cardiovascular system, immune system, nervous system, voice, blood and blood-forming tissues, metabolism/homeostasis, breast, growth, constitutional symptoms, digestive system, neoplasm, thoracic cavity, prenatal development or birth, eye, ear, skeletal system, limbs, connective tissue, endocrine system, integument). These categories are grouped into the Phenotypic Abnormalities sub-ontology. It comprises 5661 phenotypes, among which 3802 are leaves.

Gene-disease associations are extracted from our curated database, eDGAR [14] (August 2017 release), which collects information from OMIM, Humsavar and ClinVar.

### Enrichment analysis

For each group of genes associated to the same phenotype, the functional characterization is performed with NET-GE [15], an algorithm for standard and network-based gene enrichment analysis that includes the annotations derived from GO, KEGG and Reactome pathways. Briefly, it relies on the STRING Human Interactome [16], to build function-specific modules of interacting genes, starting from genes/proteins annotated with a given term. Then, given a list of genes/proteins, the over-represented modules are retrieved and scored with a *p*-value computed with an exact Fisher test and corrected with the Bonferroni procedure. A significance threshold of 0.05 has been considered.

The Information Content (IC) is computed for each GO term, KEGG and REACTOME pathways, adopting the following equation:

1 $$ {IC}_{term}=-{\mathit{\log}}_2\left(\frac{N_{term}}{N_{root}}\right) $$

where N_term_ is the number of human genes endowed with the particular GO, KEGG or REACTOME term and N_root_ is the number of human genes annotated in the ontology. IC lower bound is zero; high IC values indicate that a small number of genes are annotated with a particular term in the human genome and therefore the annotation is highly informative.

For every phenotype in PhenPath, we perform the enrichment procedure via NET-GE algorithm and we report the results in the PhenPathDB webpages. Using PhenPathTOOL, the users may compare different phenotypes retrieving the enriched biological pathways shared over the phenotypes in input. For each term describing a pathway, we report the *p*-value of the significant associations to every phenotype in input.

### Blind dataset for the performance evaluation

For the evaluation of the performance of PhenPathTOOL we collected a dataset of phenotype-disease-gene associations from Orphanet, a resource for rare diseases with high-quality information [37]. In Orphanet (release Dec 2018), 3765 diseases are associated both with HPO phenotype terms and genes. We filtered out all diseases mapped to OMIM and therefore used for the implementation of PhenPathDB, retaining 550 Orphanet diseases. We then collected diseases associated with 2 or more HPO phenotypes, ending up with 87 diseases, which form a blind set for testing PhenPathTOOL. For each disease, we entered in PhenPathTOOL the associated HPO phenotypic terms and we retrieved the list of genes they share. We compare these proposed genes with the genes reported by Orphanet for the disease. The evaluation dataset is provided as supplementary material (see Additional file 1).

## Additional file

Additional file 1: External dataset used for PhenPath evaluation. We estimate the performance of PhenPathTOOL in retrieving relevant associations between groups of co-occurring phenotypes and possible causative genes, collecting from Orphanet [37] a blind set consisting of 87 diseases, not included in OMIM nor, consequently, used to build PhenPathDB (see Methods section 5.3 for further details on the dataset). (TSV 23 kb)

## Abbreviations

BP: Biological Process
CC: Cellular ComponentCC
GO: Gene Ontology
HPO: Human Phenotype Ontology
IC: Information Content
MF: Molecular function
